# Level of awareness and utilization of insecticide-treated bed nets among medical students as measures for reducing malaria episodes

**DOI:** 10.1038/s41598-024-60523-7

**Published:** 2024-05-02

**Authors:** Mary Isioma Ofili, Bartholomew Chukwuebuka Nwogueze

**Affiliations:** 1 Department of Nursing Science, Delta State University, Abraka, Delta State Nigeria; 2 Department of Physiology, Delta State University, Abraka, Delta State Nigeria

**Keywords:** Malaria, Awareness, Utilization, Insecticide-treated nets, Physiology, Environmental sciences

## Abstract

This study examined the level of awareness and utilization of insecticide-treated bed nets among medical students as measures for reducing malaria episodes in Delta State University, Abraka. It was a descriptive study with objectives and research questions formulated to achieve the study design. A sample size of 200 male and female students resident in the campus hostels were selected using random sampling technique. A self-structured questionnaire was designed and administered to the study participants, however, only 148 copies of the questionnaires were successfully retrieved and used for the study. Data generated were subjected to quantitative statistical analysis for frequencies, percentages, average mean and Chi-square testing. Findings revealed that the level of awareness was significantly associated with the role of health workers in the distribution of insecticide-treated bed nets in Delta State University, Abraka, although, factors hindering health workers from distributing insecticide-treated bed nets were identified. There was significant difference between perception of medical students and the utilization of insecticide-treated bed nets on risk of malaria spread. In addition, there was significant difference between the benefits of using insecticide-treated bed nets and the prevention and control of malaria. We therefore conclude that regular utilization of insecticide-treated bed nets due to adequate awareness eliminates contact with mosquitoes and prevents transmitting vectors of malaria from having contact with the users of insecticide-treated bed net. Massive health education campaign is recommended to further scale up the awareness and effective utilization of insecticide-treated bed nets towards prevention and control of malaria bites among students in Delta State University, Abraka.

## Introduction

Malaria is one of the public health problems and life-threatening disease assuming wider dimension globally and has been reported to be the leading cause of death especially among children^1^. According to Antonio-Nkondjio et al*.*^2^ the heavy burden of malaria is testimony to the ability of natural breeding sites to sustain vector populations especially in rural Africa. Obviously, there is great diversity in breeding site and species distribution of anopheles mosquito egg, larvae, and pupae composition which varies considerably and are most likely to be found in permanent, shallow, sunlit pools of water, seepages, springs, streams, river bed and river streams with the most common been ponds and swamps. There is a great variation in breeding site preference among anopheles’ species, such as; *An. Stephensi* which is common in urban India, *An. funestus and An. Arabiensis* are the major malaria vector in East Africa while *An. darling, An. epirotus* and *An. gambiae* are primary vector in Sub-Sahara Africa and Asia^3^.

The serious burden of malaria as an acute febrile sickness common in children and in pregnancy are attributed to the sequestration of plasmodium parasites in the placenta, leading to impeded trans-placental nutrient transport. With complications of malaria in pregnancy and children resulting in maternal anemia, compromised foetal growth, low birth weight (LBW), preterm delivery, and fetal intrauterine deaths^4,5^. This calls for several efforts targeted towards malaria prevention, control and campaigns such as; Roll Back Malaria (RBM) Program and World Health Organization (WHO) new global guidelines, relying mostly on indoor residual spraying (IRS), insecticide-treated bed nets (ITNs), and effective treatment with antimalarial drugs as crucial strategies for reducing maternal, perinatal and children morbidity and mortality related to malaria^6,7^.

Insecticide-treated Nets (ITNs) is a type of medically treated net which offers protection against mosquitoes, flies, and other insects, and thus against the disease they may transmit^8^. ITNs provides protection for those who sleep under them while also killing any mosquitoes that come in contact with them, minimizing the incidence, density, proportions, and transmission potential of malaria spread by female anopheles mosquitoes, thus, contributing to control and prevention mosquito bites and the magnitude and burden of malaria transmission episodes that is linked to reduction of morbidity and mortality globally^8^. ITN users are protected by the netting's physical barrier, but as mosquitoes are diverted away from those wearing non-lethal bed nets, non-users may experience an increase in bite rates^1^. This technique (ITNs) offers some protection to other people, including those who are sleeping in the same room but outside the net. However, in some situations, transmission potential of malaria may become worse after bed nets lose their insecticidal effects^9^.

The awareness and knowledge of the citizenry towards the use of insecticide-treated bed nets campaigns and sensitization in the control of mosquito bite and malaria parasite exposure has been the major effective and reliable tool for mitigating the overall spread and effect of malaria around the world^10^. However, it is important to note that it has not produced the desired results especially in rural areas owing to a multiplicity of factors which act as impediments to the success of this noble idea^11^. Some of these factors are blamable on the rate at which those at the hem of affairs delay to take adequate actions by way of remediating the ugly situation. Factors militating against the use of insecticide-treated bed nets include; cultural, behavioural and demographic factors, ethnicity, accessibility, gender relations and seasonality of malaria^11,12^. Many nations of the world today are ravaged by malaria and the need for the provision of insecticide-treated nets becomes imperative^10,13^. Though, there are relentless campaigns and sensitization programmes to curb the spread and burden of malaria infection all over the world^14^. The situation in Nigeria about malaria is worsening every day since its discovery, considering that an estimated 68million cases of malaria-related deaths are recorded yearly^15^. However, a number of studies have found that insecticide-treated nets provides varying degrees of protection against malaria morbidity, anemia and Low Birth Weight (LBW)^16–18^.

The outcomes and associated outputs of the present study will enable the management of Delta State University to inform policies in collaboration with health policy executors on how to equip all students with ITNs in order to realize the set goals and objectives of reducing malaria episodes. The spread of mosquito nets or bed nets treated with insecticides such as permethrin or deltamethrin has proven to be a highly successful strategy for preventing malaria episodes^11^. In endemic locations, it is also one of the most affordable ways to provide personal protection, however, there is a serious problem of efficacy with pyrethroid only treated nets that are ubiquitous in Africa in this era of widespread pyrethoid resistance to malaria vector control^19,20^. It is pathetic to observe that the effects of malaria infestation have been great in riverine areas, especially in the Niger Delta region and specifically in the locality of Abraka, were Delta State University multi-campuses are situated with scores of malaria cases recorded within the university health facilities daily^21,22^. This therefore informed the decision to assess the awareness and level of utilization of insecticide-treated bed nets in the control and prevention of malaria among medical students of Delta State University, Abraka, while appraising the strategies that can be put in place in order to prevent the situation from getting out of control.

## Materials and methods

### Study site

This study was conducted in the Faculty of Basic Medical Sciences of Delta State University Abraka. The Faculty trains medical students under five (5) Departments namely; Anatomy and Cell Biology, Medical Biochemistry, Nursing Science, Pharmacology and Human Physiology respectively. The rational for choosing this site was because the concerned medical student hostels must reside in the school hostel in Campus 1, 2 and 3 respectively for ease of identification, and administration of questionnaire.

### Study design

A descriptive survey design was employed in the study to ascertain the level of awareness and utilization of Insecticide-Treated Bed Nets among Medical Students in Delta State University, Abraka. The design involves the systematic collection, analysis and presentation of data to give a clear picture of the present situation. It provides an accurate account or the characteristics of a particular individual use and situation. It studies relationships between variables in the present, describes events, determines their frequency and helps to discover new meaning. The rationale behind the use of this design is that the event to be investigated had already occurred.

### Study participants

The participants for the study comprised all the male and female students from 100 to 400 level that cuts across the five Departments in the Faculty of Basic Medical Sciences but are resident in the school hostels across the three (3) main campuses of Delta State University, Abraka. A total of 400 students constituted the target population. In order to select a fairly representative sample from the population, Slovin’s formula for sample size determination was used for the study. It has the formula:

$$n=\frac{N}{1+N{\left(e\right)}^{2}}$$

$$n=\frac{400}{1+400{\left(0.05\right)}^{2}}$$

$$\frac{400}{2} = 200$$

where; *n* = desired sample size; N = population of the study (400); e = Tolerable Error (0.05) and 1 = Theoretical constant. Hence, a sample size of 200 medical students were selected from a target population of 400 using simple random sampling technique. To calculate the sample size of respondents for each Department from the Faculty, the formula below was used;

$$r=\frac{RPS}{N} x n$$

where; *r* = respondent from each Department, *RPS* = respondent sample size; N = population of the study and *n* = desired sample size.

### Inclusion and exclusion criteria

Only apparently healthy male and female students that are resident in the campus hostel and falls between the ages of 18–45 years from the Faculty of Basic Medical Sciences were considered in the study. The category of subjects not included in the study were students staying off-campus and those that are not from the Faculty, in addition to students that does not have prior knowledge of insecticide-treated bed nets.

### Data collection tool

A self-administered questionnaire was formed and utilized as the research instrument for the study. The questionnaire contained a structured 16 items aimed at eliciting responses from the respondents. The respondents are offered a choice of alternatives using a modified Likert 4-point scale of Strongly Agree, Agree, Disagree and Strongly Disagree. The instrument was validated by two Research experts in measurement and evaluation. The instrument was vetted in terms of relevance appropriateness, clarity and comprehensiveness of the content. Based on their corrections and comment, modifications were made which resulted in the final draft questionnaire used in this study.

### Method of data collection

Two hundred (200) copies of the data collection tool were administered by the researcher with the assistance of the heads of the various Departmental class representatives duly trained for purpose of this research. After two weeks following administration, 148 copies completed questionnaires were successfully collected, coded and subjected to statistical analysis. The mutilated and uncompleted questionnaires were voided and rejected.

### Method of data analysis

Data collected were analyzed for descriptive statistics using frequency counts, percentages and the mean score statistical method. The analyses were presented in tables based on the generated research questions and formulated research hypotheses. A range of mean was determined by summing the average responses on the 4-point Likert scale (4 = strongly agree, 3 = agree, 2 = disagree and 1 = strongly disagree), then divide by the total number of respondents and this was used as the interpretative norm to determine or ascertain the level of acceptance or rejection. The interpretative norm included; 1.0–2.44 (Reject); 2.50 and above (Accept). Chi-square test was the inferential statistics used to test the formulated null hypotheses. A p-value < 0.05 level was considered significant.

### Consent and ethical clearance

An informed consent was obtained from all subjects and/or their legal guardian(s) prior to the study. All experimental protocols were approved by a named institutional ethical committee with permit number: REC/FBMS/DELSU/23/167. All procedures were performed in accordance with relevant guidelines.

## Results

Table 1 presents data on demographic information of respondents. It was observed that 89 (60.1%) of the study participants were female medical students, while 59 (39.9%) were males. Our data shows that the highest responses based on females were from Nursing Science, with the least been from Pharmacology. For male participants, 17% were from Pharmacology as majority and 3.4% were from Nursing Sciences as the least. The age distribution of respondents indicates that 29 (19.6%) of the respondents were within the age bracket of < 15 years of age. The highest participants with a frequency of 68 representing 45.9% were within the age brackets of 16–20 years of age. More so, 37 (25%) of the sample were within the age brackets of 21–25 years, whereas, the remaining 14 (9.5%) respondents were within the age bracket of 26 years and above. There is a clear indication that majority of the participants were between the age brackets of 16–20 years irrespective of their departments.

**Table 1:** Socio-demographic information based on departments of respondents.

Variables	Anatomy and cell biology		Medical biochemistry		Nursing science		Pharmacology		Human physiology		Total	
	*F*	*%*	*F*	*%*	*F*	*%*	*F*	*%*	*F*	*%*	*F*	*%*
Total	30	20.2	28	18.9	30	20.2	30	20.2	30	20.2	148	100
Gender												
Female	19	12.8	18	12.2	25	16.8	13	8.8	14	9.4	89	60.1
Male	11	7.4	10	6.7	5	3.4	17	11.4	16	10.8	59	39.2
Age												
< 15 years	4	2.7	8	5.4	6	4.0	5	3.3	6	4.0	29	19.6
16–20 years	16	10.8	12	8.1	13	8.8	10	6.8	17	11.4	68	45.9
21–25 years	9	6.0	6	4.0	5	3.4	12	8.1	5	3.4	37	25.0
26 years and above	1	0.6	2	1.3	6	4.0	3	2.0	2	2.4	14	9.5
Marital status												
Married	3	2.0	2	1.3	8	5.4	1	0.6	5	3.4	19	12.8
Single	25	16.9	23	15.6	21	14.2	29	19.6	20	13.5	118	79.7
Divorced	0	0.0	0	0.0	0	0.0	0	0.0	1	0.6	1	0.7
Others	2	1.3	3	2.0	1	0.6	0	0.0	4	2.7	10	6.8
Religion												
Christianity	26	17.6	24	16.2	25	16.9	24	16.2	28	18.9	127	85.8
Islam	0	0.0	0	0.0	0	0.0	2	1.3	0	0.0	2	1.4
ATR	3	2.0	4	2.7	2	1.3	4	2.7	2	1.3	15	10.1
Others	1	0.6	0	0.0	3	2.0	0	0.0	0	0.0	4	2.7
Level of awareness of ITN utilization												
Aware	28	18.9	25	16.9	30	20.2	27	18.2	30	20.2	140	94.6
Not aware	2	1.3	3	2.0	0	0.0	3	2.0	0	0.0	8	5.4
Possession of ITNs among students												
With ITNs	9	6.0	8	5.4	24	16.2	13	8.8	12	8.1	66	44.6
Without ITNs	21	14.2	20	13.5	6	4.0	17	11.4	18	12.1	82	55.4
Rate of utilization of ITN among students												
Always	10	6.7	5	3.4	18	12.1	11	7.4	12	8.1	56	37.8
Sometimes	18	12.2	20	13.5	12	8.1	17	11.5	15	10.1	82	55.4
Never	2	1.3	3	2.0	0	0.0	2	2.3	3	2.0	10	6.8

**Key:** *ITN* insecticide-treated nets, *F* frequency, *%* percentage, *ATR* African traditional religion.

On the basis of marital status, 118 (79.7%) respondents were single, 19 (12.8%) were married, 10 (6.8%) choose other marital status. The highest responses for married participants were from Nursing Science and the least were from Medical Biochemistry. Conversely, majority of the single participants were from Pharmacology and the least single participants were from Physiology. In a nutshell, majority of the students were unmarried, notwithstanding their respective departments. Similarly, based on religion, 127 (85.8%) of the students practiced Christianity, 2 (1.4%) practiced Islam, 15 (10.1%) practice African Traditional Religion, and the remaining 4 (2.7%) practiced other religion, thus, majority of the students were Christians irrespective of their departments. In the present study, the awareness level for ITNs revealed that 140 (94.6%) were adequately informed about the utilization of ITNs when compared to the remaining 8 (5.4%) that were not aware. This observation of positive awareness level was recorded across all Departments. Responses on the basis of possession of ITNs revealed that only 66 (44.6%) of the participants owned ITNs when compared to 82 (55.4%) without ITNs. Nursing Science students possessed the highest ITNs while the least was observed among students from Anatomy & Cell Biology. On the rate of utilization of ITNs, it was shown that 82 (55.4%) used the nets sometimes, while 56(37.8%) used the nets always. However, only 10 (6.8%) had never utilized ITNs.

### Answering of research questions

***RQ1:*** *What is the level of awareness on the role of health workers in the distribution of insecticide-treated bed nets in Delta State University, Abraka?*

Table 2 shows the level of awareness on the role of health workers in the distribution of insecticide-treated bed nets among medical students. The mean score of 2.91 suggests that there is adequate awareness on adequate campaign and programme conducted by health workers to promote distribution of insecticide-treated bed net. Also, a mean score of 3.35 respondents agreed that the respondents were aware of the efforts of health workers role in providing solution to the few houses where there are insecticide-treated bed nets with complaints of inconveniences. A mean score of 3.05 respondents agreed that they are aware of the attitude of some health personnel selling insecticide-treated bed nets meant to be distributed and as a result those who do not have become discouraged from having one. Similarly, a mean score of 2.91 respondents are of the opinion that they are aware of factors hindering health workers from distributing insecticide-treated bed net. Finally, a mean score of 2.85 respondents are of the opinion that they are aware of the role of University health center in sensitizing students on the risk factor of not using insecticide-treated bed nets. Considering that the aggregate mean of 3.02 is higher than the criterion mean of 2.50, we conclude that the level of awareness was significantly associated with the role of health workers in the distribution of insecticide-treated bed nets in Delta State University, Abraka to very high extent.

**Table 2:** Level of awareness on the role of health workers in the distribution of insecticide-treated bed nets.

S/N	Statements	SA 4	A 3	D 2	SD 1	$\mathop x\limits^{\vee }$	Decision
1	Awareness on adequate campaign and programme conducted by Health workers to promote distribution of insecticide-treated bed net	61	37	26	24	2.91	Accepted
2	Awareness of the efforts of health workers role in providing solution to the few houses where there are insecticide-treated bed nets with complaints of inconveniences	80	48	13	7	3.35	Accepted
3	Awareness of the attitude of some health personnel selling insecticide-treated bed nets meant to be distributed and as a result those who do not have become discouraged from having one	63	46	23	16	3.05	Accepted
4	Awareness of factors hindering health workers from distributing insecticide-treated bed net	45	56	36	11	2.91	Accepted
5	Awareness of the role of University health center in sensitizing students on the risk factor of not using insecticide-treated bed nets	42	54	40	12	2.85	Accepted
Grand mean						3.02	Adopted

**Keys**: $\mathop x\limits^{\vee }$ mean, *SA* strongly agree, *A* agree, *D* disagree, *SD* strongly disagree.

***RQ2:*** *What is the perception of medical students on the utilization of insecticide-treated bed nets on risk of malaria in Delta State University, Abraka?*

Table 3 shows perception on the utilization of insecticide-treated bed nets on risk of malaria. The mean score of 3.34 suggests that those who have insecticide-treated bed nets do not use them as a result of heat have chanced of been infected with malaria. The respondents as reflected by the mean score of 2.86 indicated that some do not use insecticide-treated bed nets despite the risk of malaria because they are inconvenienced as a result of having contacts with the user while asleep. A mean score of 2.80 accepted that some prefer having malaria than using insecticide-treated bed nets because they are afraid of possible reaction with the insecticide. Similarly, a mean score of 3.10 indicated that the use of insecticide-treated bed nets helps to prevent mosquito bites and spread of malaria burden. Finally, a mean score of 2.72 respondents agreed that the use of insecticide-treated bed nets is ceremonious by some people owing to a sense of pride and not necessary because of the risk of malaria. Considering that the aggregate mean of 2.96 is higher than the criterion mean of 2.50, we conclude that the perception on the utilization of insecticide-treated bed nets on risk of malaria among medical students in Delta State University, Abraka were adequately adopted to a large extent.

**Table 3:** Perception of medical students on the utilization of insecticide-treated bed nets on risk of malaria.

S/N	Statement	SA 4	A 3	D 2	SD 1	$\mathop x\limits^{\vee }$	Decision
1	Those who have insecticide-treated bed nets do not use them as a result of heat have chanced of been infected with malaria	80	43	20	5	3.34	Accepted
2	Some do not use insecticide-treated bed nets despite the risk of malaria because they are inconvenienced as a result of having contacts with user while asleep	52	49	21	26	2.86	Accepted
3	Some prefer having malaria than using insecticide-treated bed nets because they are afraid of possible reaction with the insecticide	46	50	28	24	2.80	Accepted
4	The use of insecticide-treated bed nets helps to prevent mosquito bites and spread of malaria burden	73	36	20	19	3.10	Accepted
5	The use of insecticide-treated bed nets is ceremonious by some people owing to a sense of pride and not necessary because of the risk of malaria	45	46	28	29	2.72	Accepted
Grand mean						2.96	Adopted

**Keys**: $\mathop x\limits^{\vee }$ mean, *SA* strongly agree, *A* agree, *D* disagree, *SD* strongly disagree.

***RQ3:*** *knowledge of medical students on the benefits of using insecticide-treated Bed nets in the Prevention and Control of Malaria in Delta State University, Abraka*

Table 4 shows the benefits of using insecticide-treated bed nets in the prevention and control of malaria. The mean score of 2.86 suggested that majority of the respondents believes that regular use of insecticide-treated bed nets eliminates contact with mosquitoes. The mean score of 3.34 suggest that transmitting vectors of malaria are prevented from having contact with the users of insecticide-treated bed net. The mean score of 3.29 suggest that avoidance of contact with the insecticide-treated bed nets prevented mosquitoes that have the ability to bite from outside the net. The mean score of 2.87 suggest that due to the netting size (mesh), mosquitoes are barricaded from having contact with the user while asleep which by extension could prevent risk of been infected with malaria. Finally, a mean score of 3.00 respondents agreed that there is adequate level of awareness campaign and the sensitization on insecticide-treated bed nets. Considering that the aggregate mean of 3.07 is higher than the criterion mean of 2.50, we conclude that the use of insecticide-treated bed nets in the prevention and control of malaria among medical students in Delta State University, Abraka was adopted to a very large extent.

**Table 4:** Benefits of using insecticide-treated bed nets in the prevention and control of malaria.

S/N	Statement	SA 4	A 3	D 2	SD 1	$\mathop x\limits^{\vee }$		Decision
1	Regular use of insecticide-treated bed nets eliminates contact with mosquitoes	47	61	16	24	2.89		Accepted
2	Transmitting vectors of malaria are prevented from having contact with the users of insecticide-treated bed net	82	46	9	11	3.34		Accepted
3	Avoidance of contact with the insecticide-treated bed nets prevented mosquitoes that have the ability to bite from outside the net	78	42	12	16	3.23		Accepted
4	Due to the netting size (mesh) mosquitoes are barricaded from having contact with the user while asleep which by extension could prevent risk of been infected with malaria	48	55	23	22	2.87		Accepted
5	Adequate level of awareness campaign and the sensitization on insecticide-treated bed nets	56	49	30	13	3.00		Accepted
Grand mean							3.07	Adopted

**Keys**: $\mathop x\limits^{\vee }$ mean, *SA* strongly agree, *A* agree, *D* disagree, *SD* strongly disagree.

### Testing of research hypotheses

***H1:*** *There is no significant difference between the level of awareness of medical students and the role of health workers in the distribution of insecticide-treated bed nets*

Table 5 represents the statistics for null hypothesis I using Chi-square statistical analysis for categorical data. The analysis procedure involved pooling respondents’ scores (responses) to the 4-point Likert scale and analyzing for observed and expected frequency for each items measured. Since the calculated X^2^ value of 49.43 is greater than $\ge$ the table X^2^ value of 16.92 with a degree of freedom of 12, hence, the null hypothesis earlier stated is rejected and the alternate hypothesis is accepted, we therefore conclude that there is significant difference between the level of awareness of medical students and the role of health workers in the distribution of insecticide-treated bed nets in Delta State University, Abraka.

**Table 5:** Testing of null hypothesis I.

SA		A		D		SD		Cal X^2^	Tab X^2^	*df*	P-value
O	E	O	E	O	E	O	E				
61	(58.2)	37	(48.2)	26	(27.6)	24	(4.9)	49.43	16.92	12	0.001
80	(58.2)	48	(48.2)	13	(27.6)	7	(4.9)				
63	(58.2)	46	(48.2)	23	(27.6)	16	(4.9)				
45	(58.2)	56	(48.2)	36	(27.6)	11	(4.9)				
42	(58.2)	54	(48.2)	40	(27.6)	12	(4.9)				
291		241		138		70					

**Decision**: Hypothesis I is statistically significant at ***p < 0.0001 (row = 5, columns = 4).

**Key**: *O* observed frequency, *E* expected frequency, *df* degree of freedom, *Calculated ****X***^***2***^ calculated chi square, *Tab ****X***^***2***^ tabulated chi square.

***H2:**** There is no significant difference between the perception of medical students and the utilization of insecticide-treated bed nets* on risk of malaria

Table 6 represents the statistics for null hypothesis II using Chi-square statistical analysis for categorical data using a 4-point Likert scale in analyzing for observed and expected frequency for each items measured. Considering that the calculated X^2^ value of 40.96 is less than $\ge$ the table X^2^ value of 16.92 with a degree of freedom of 12, hence, the null hypothesis II earlier stated is rejected and the alternate hypothesis is rejected, thus, we conclude that there is significant difference between the perception of medical students and the utilization of insecticide-treated bed nets on risk of malaria in Delta State University, Abraka.

**Table 6:** Testing of null hypothesis II.

SA		A		D		SD		Cal X^2^	Tab X^2^	*df*	P-value
O	E	O	E	O	E	O	E				
80	(59.2)	43	(44.8)	20	(23.4)	5	(20.6)	40.96	16.92	12	0.000
52	(59.2)	49	(44.8)	21	(23.4)	26	(20.6)				
46	(59.2)	50	(44.8)	28	(23.4)	24	(20.6)				
73	(59.2)	36	(44.8)	20	(23.4)	19	(20.6)				
45	(59.2)	46	(44.8)	28	(23.4)	29	(20.6)				
296		224		117		103					

**Decision:** Hypothesis II is not statistically significant at p < 0.001 (row = 5, columns = 4).

**Key**: *O* observed frequency, *E* expected frequency, *df* degree of freedom, *Calculated ****X***^***2***^ calculated chi square, *Tab ****X***^***2***^ tabulated chi square.

***H3:*** *There is no significant difference between the benefits of using insecticide-treated bed nets and the prevention and control of malaria*

Table 7 represents the statistics for null hypothesis III using Chi-square statistical analysis for categorical data. The analysis procedure involved pooling respondents’ scores (responses) to the 4-point Likert scale and analyzing for observed and expected frequency for each items measured. Since the calculated X^2^ value of 45.82 is greater than $\ge$ the table X^2^ value of 16.92 with a degree of freedom of 16, hence, the null hypothesis earlier stated is rejected and the alternate hypothesis is accepted, we therefore conclude that there is significant difference between the benefits of using insecticide-treated bed nets and the prevention and control of malaria in Delta State University, Abraka.

**Table 7:** Testing of null hypothesis III.

SA		A		D		SD			Cal X^2^	Tab X^2^	*df*	P-value
O	E	O	E	O	E		O	E				
47	(62.2)	61	(50.6)	16	(18)		24	(17.2)	45.82	16.92	12	0.001
82	(62.2)	46	(50.6)	9	(18)		11	(17.2)				
78	(62.2)	42	(50.6)	12	(18)		16	(17.2)				
48	(62.2)	55	(50.6)	23	(18)		22	(17.2)				
56	(62.2)	49	(50.6)	30	(18)		13	(17.2)				
311		253		90			86					

**Decision:** Hypothesis III is statistically significant at ***p < 0.001 (row = 5, columns = 4).

**Key**: *O* observed frequency, *E* expected frequency, *df* degree of freedom, *Calculated ****X***^***2***^ calculated chi square, *Tab ****X***^***2***^ tabulated chi square.

## Discussion

The analysis of findings as regards perception of respondents on level of awareness on the role of health workers in the distribution of insecticide-treated bed nets revealed that majority accepted that there is adequate awareness on adequate campaign and programme conducted by health workers to promote distribution of insecticide-treated bed net, health workers play important role in providing solution to the few houses where there are insecticide-treated bed nets with complaints of inconveniences. Although, it was noted that some health personnel sell insecticide-treated bed nets meant to be distributed and as a result those who do not have are discouraged from having. In addition, factors hindering health workers from distributing insecticide-treated bed nets were identified and that University health center always sensitizes the students on the risk factor of not using insecticide-treated bed net. This finding is consistent with previous studies who reported that the level of awareness on the distribution of insecticide-treated bed nets is dependent on the level of sensitization of the people^23–25^.

Research question two sought to find out the perception of medical students on the utilization of insecticide-treated bed nets. The analysis of findings revealed that those who have insecticide-treated bed nets do not use them as a result of heat have chanced of been infected with malaria while some do not use insecticide-treated bed nets despite the risk of malaria because they are afraid of possible reaction with the insecticide notwithstanding that it helps to prevent mosquito bites and spread of malaria burden. Similarly, it was adopted that the utilization of insecticide-treated bed nets is ceremonious by some people owing to a sense of pride and not necessary because of the risk of malaria. This is contrary to the opinion of Cohen and Dupas^26^ who agreed that the use of insecticide-treated bed nets by the people of Southern Sudan has contributed immensely in the prevention and control of malaria in the area and therefore advocated for the use of the net. Although, their works was not done among medical students in Nigeria. According to Chukwuocha et al.^27^ the low level of actual use of ITNs could be attributed to poor knowledge, attitude and practices regarding the use of ITNs as well as socioeconomic and cultural factors like; poor or inconvenient accommodation to hang the nets.

The analysis of findings for research question three on the perception of respondents on the benefits of using insecticide-treated bed nets in the prevention and control of malaria among medical students in Delta State University Abraka revealed that regular utilization of insecticide-treated bed nets eliminates contact with mosquitoes and that transmitting vectors of malaria are prevented from having contact with the users of insecticide-treated bed net. More so, majority believed that avoidance of contact with the insecticide-treated bed nets prevented mosquitoes that have the ability to bite from outside the net and that due to the netting size (mesh) mosquitoes are barricaded from having contact with user while asleep which by extension prevent risk of been infected with malaria. Thus, majority accepted that there is adequate level of awareness campaign and the sensitization on insecticide-treated bed nets. These view are supported by the study of Yassin et al*.*^28^ who reported that a plethora of factors influencing the utilization of insecticide-treated bed net. The author maintained increasing level of knowledge on the proper usage of ITNs increases its efficiency and this significantly increases the confidence of pregnant women to use ITNs for prevention of malaria during their pregnancies.

## Conclusion

The present study has revealed that the level of awareness was significantly associated with the role of health workers in the distribution of insecticide-treated bed nets. There was significant difference between perception of medical students and the utilization of insecticide-treated bed nets on risk of malaria spread. In addition, there was significant difference between the benefits of using insecticide-treated bed nets and the prevention and control of malaria. Therefore, we conclude that regular utilization of insecticide-treated bed nets due to adequate awareness eliminates contact with mosquitoes and prevents transmitting malaria vectors from having contact with the users of insecticide-treated bed net. Hence, insecticide-treated bed nets awareness programmes should be taken to the student hostels both on-campus and off-campuses in order to create more awareness on the benefit of utilization of ITN. This will enhance the realization of the goals and objectives of reducing malaria episodes in Delta State University, Abraka.

## Data Availability

The datasets generated and/or analyzed during the study will be made available on reasonable request from the corresponding author.
